# Bioluminescent *Mycobacterium abscessus* in *Galleria mellonella*: a preclinical screening tool for antimycobacterial compounds

**DOI:** 10.3389/fcimb.2026.1817296

**Published:** 2026-05-08

**Authors:** Tine Van Win, Wannes Brangers, Agustin Reséndiz Sharpe, Gert-Jan Wijnant, Emmanuel André, Greetje Vande Velde

**Affiliations:** 1 Laboratory of Clinical Microbiology, Department of Microbiology, Immunology and Transplantation, KU Leuven, Leuven, Belgium; 2 Biomedical MRI, Department of Imaging and Pathology, KU Leuven, Leuven, Belgium; 3 Department of Laboratory Medicine, UZ Leuven, Leuven, Belgium

**Keywords:** antimycobacterial agents, bioluminescence imaging, drug screening, *in vivo* infection model, mycobacterial infection, *Mycobacterium abscessus*, non-mammalian model

## Abstract

*Mycobacterium abscessus* (Mab) is an emerging pulmonary pathogen with extensive drug resistance, resulting in prolonged multidrug therapy, severe side effects, and cure rates below 50%. The absence of a reliable immunocompetent mouse model and the poor translation of *in vitro* drug activity to mammalian *in vivo* efficacy hinder preclinical progress. To address this gap, we developed a novel *Galleria mellonella* infection model for Mab, combined with bioluminescence imaging (BLI), enabling longitudinal monitoring and quantification of mycobacterial burden. Two double-reporter strains were assessed: a firefly luciferase*-*expressing strain requiring exogenous luciferin, and an autonomously luminescent Lux strain. Both generated stable signals suitable for longitudinal *in vitro* and *in vivo* imaging, with improved *in vivo* detectability of the luciferase-expressing strain based on its characteristic red-shifted emission spectrum. BLI provided sensitive, early quantification of mycobacterial load and treatment responses, outperforming traditional larval endpoints. This model supports early detection of dose-dependent treatment effects and enables real-time monitoring, thereby highlighting the value of *G. mellonella* as a rapid, cost-effective complementary preclinical model for screening novel antimycobacterial compounds.

## Graphical Abstract

**Figure:**
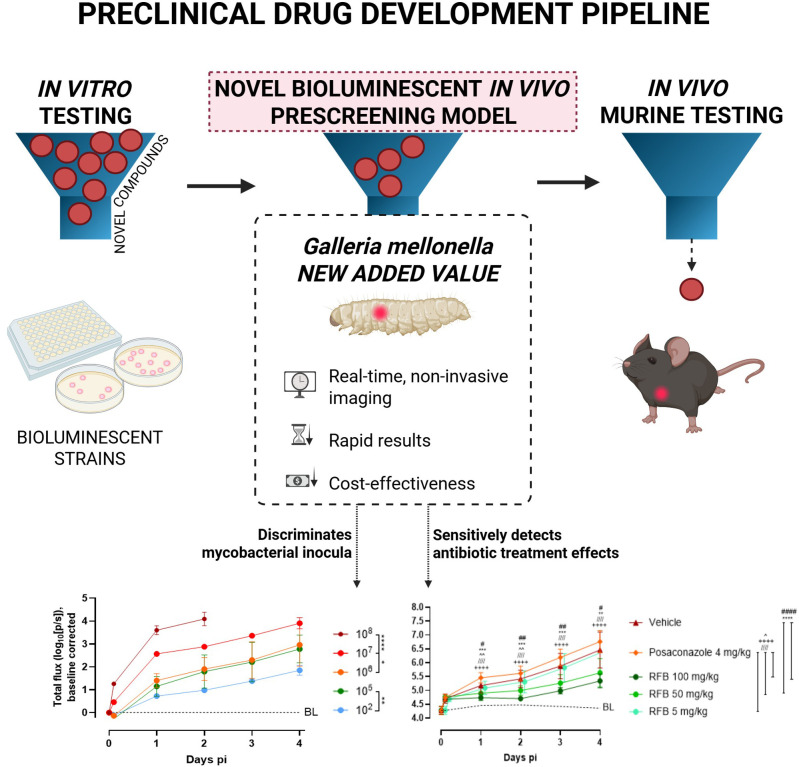

## 1 Introduction

*Mycobacterium abscessus* (Mab) is a rapidly growing mycobacterial species and an emerging global pathogen responsible for difficult-to-treat infections. Mab most commonly causes non-tuberculous mycobacterial pulmonary disease (NTM-PD), but can also lead to skin, soft-tissue, and ocular infections (Riva et al., 2020; Kumar et al., 2022; Dedrick et al., 2023). It poses a significant clinical challenge due to intrinsic resistance to most antibiotics, and the increasing emergence of acquired resistance, resulting in cure rates below 50% (Bento et al., 2024). NTM-PD is considered a neglected disease and occurs more frequently in individuals with underlying lung diseases, such as cystic fibrosis (CF), as well as in immunocompromised patients (To et al., 2020; Troian et al., 2023). Current treatment regimens are lengthy and involve multiple antibiotics that often lead to severe side effects and poor patient compliance (Bento et al., 2024). Clinical outcomes remain poor, with reported mortality rates ranging from 7.6% to 15.7% and culture conversion achieved in fewer than half of patients across cohorts. Beyond the clinical burden, Mab infections also impose a growing economic impact, exemplified by substantially higher direct healthcare costs reported for NTM-PD patients in Germany (Ballarino et al., 2009; Strollo et al., 2015; Diel et al., 2017; Sfeir et al., 2018; Winthrop et al., 2020; Chang et al., 2024). These challenges highlight the urgent need for novel antimycobacterial therapies.

Yet, drug development is slowed by limitations in current preclinical models. Many compounds with strong *in vitro* activity fail to translate into mammalian *in vivo* efficacy, partly due to the lack of efficient intermediate models for early efficacy screening (Meir et al., 2018; Hosoda et al., 2020; Theuretzbacher et al., 2020). Alternative models such as *Drosophila melanogaster* and zebrafish have been explored, but their cost, limited accessibility, and inability to thrive at human physiological temperatures (>30 °C) restrict broader use (Li et al., 2013; Meir et al., 2018). In contrast, *Galleria mellonella* larvae have emerged as an intermediate model for antibacterial drug testing due to their low cost, ease of handling, absence of major ethical constraints, and suitability for high-throughput screening (Vanhoffelen et al., 2023). This model has proven valuable for studying *M. abscessus* infection and treatment responses, establishing its feasibility as a host for preclinical investigation (Meir et al., 2018). Incorporating *G. mellonella* into the drug development pipeline may accelerate progress, reduce costs, and minimize mammal use in line with the 3Rs principle (Asai et al., 2023).

Traditional *Galleria mellonella* readouts, such as larval health, survival, and colony-forming units (CFU), are limited by interobserver variability, low sensitivity, and labor-intensive, single-endpoint readouts (Vanhoffelen et al., 2023). Additionally, despite being a rapid grower, Mab still requires up to seven days for CFU enumeration, further delaying experimental timelines (Kania et al., 2023; Biology insights, 2025). Bioluminescence imaging (BLI) offers a dynamic, noninvasive alternative, enabling real-time monitoring of bacterial burden with greater sensitivity and reduced animal use. BLI has already demonstrated superior performance in *G. mellonella* to classical readouts in models of *Cryptococcus* and *Aspergillus fumigatus*, and has also been applied in *G. mellonella* models of *M. abscessus* infection, where it has primarily been used to demonstrate model applicability (Meir et al., 2018; Vanhoffelen et al., 2023; Bento et al., 2024; Vanhoffelen et al., 2024; Biology insights, 2025).

Here, we evaluated two bioluminescent double-reporter *M. abscessus* strains, designed for *in vitro* and *in vivo* drug screening. A first strain expresses a red-shifted luciferase requiring D-luciferin substrate, offering improved *in vivo* signal penetration, while the second carries the luxCDABE operon enabling autonomous light production and simplified longitudinal imaging (Bento et al., 2024). Both strains also express the fluorescent protein mScarlet, allowing fluorescence imaging (FLI) as an alternative, however, non-viable bacteria can still emit a fluorescent signal, resulting in strong background signals and a low signal-to-noise ratio (Clinxsci; Bento et al., 2024).

In this study, we present a standardized and quantitatively validated bioluminescent *G. mellonella* infection model for *M. abscessus* as a robust and efficient complementary preclinical model for the screening of antimycobacterial compounds and drug combinations, addressing key limitations of current readouts and enabling improved compound selection prior to murine testing.

## 2 Materials and methods

### 2.1 *Mycobacterium abscessus* strains and culture

Two *Mycobacterium abscessus* strains, a firefly luciferase*-*expressing strain requiring exogenous luciferin administration (Mab fLuc), and a Lux expressing strain enabling autonomous bioluminescence, were used in this study (Mab Lux) (Bento et al., 2024). These strains were purchased from the UA group of Professor Paul Cos (Bento et al., 2024). All strains were grown on Middlebrook 7H10 agar medium containing 0.5% glycerol and 10% Oleic acid-Albumin-Dextrose-Catalase (OADC) for solid growth or in Middlebrook 7H9 broth containing 0.5% glycerol, 0.05% Tween 80, and 10% ADC for liquid growth. The strains were grown at 37 °C, with shaking at 150 RPM for liquid growth. Bacterial concentrations were estimated based on an optical density (OD_600_) to colony forming unit (CFU) correlation (CFU/mL) using a spectrophotometer (VWR, USA). Suspensions were centrifuged, resolubilized in PBS and subsequently diluted in PBS to the desired concentration. The final bacterial load was confirmed by CFU plating.

### 2.2 Galleria mellonella infection model

Healthy adult 6^th^ week instar larvae with normal movement, no melanization, weighing 300 ± 50 mg (in-house bred) were used for the experiments. The larvae were randomly assigned to experimental groups (n = 3–10 per group) and housed individually in 12-well transparent plates (Greiner, Austria). They were kept in the dark without food at 37 °C. Bacterial suspensions were injected (10 µL) into the last left proleg with a Hamilton syringe (25 µL/ga30/12.7 mm Hamilton, USA). The negative controls were sham-infected with 10 µL PBS. Larval health was assessed daily for four days post infection based on larval activity, melanization and survival (Vanhoffelen et al., 2023). A maximal score of nine, converted into a percentage of 100%, meant that the larva was completely healthy (Vanhoffelen et al., 2023). The experiments were limited to four days due to the onset of pupation. During pupation, larvae undergo physiological and metabolic reorganization, rendering health assessment and survival readouts no longer meaningful.

### 2.3 Bioluminescence imaging

To verify luminescence signal stability over time and to inform imaging parameters, sequential measurements were first performed on larvae one hour post infection, with scan durations of 10 minutes for Mab Lux and 20 minutes for Mab fLuc. These experiments demonstrated that, during the respective imaging periods, the bioluminescent signal reached and remained in a stable plateau phase. Based on this assessment, *in vivo* imaging of Mab fLuc-infected larvae was initiated five minutes after D-luciferin administration, corresponding to the onset of a stable luminescent signal. For Mab Lux, no luciferin administration was required. Spectral emission scans ranging from 500 to 840 nm were performed on live larvae one hour post infection, infected with 10^7^ Mab fLuc and 10^8^ Mab Lux, to assess strain-specific emission spectra. To verify the relative mycobacterial load of the inoculum, *in vitro* BLI was performed on the inoculum and *ex vivo* BLI on larval homogenates following infection. For *ex vivo* BLI, individual larvae were homogenized in 1 mL 7H9 medium using a tissue homogenizer (Tissue Master homogenizer; Omni International, USA). Ten-fold serial dilutions of both inoculum and homogenates were prepared in a black 96-well plate with a transparent bottom (Thermo Scientific, USA). For the Mab fLuc strain, 10% D-luciferin sodium salt (1.25 mg/mL in PBS; GoldBio, USA) was added to both inoculum and homogenates. *In vivo* BLI was performed on live larvae one hour post infection and daily up to four days post infection to confirm relative mycobacterial cell count, discrimination between different inocula sizes, and treatment effects. Accordingly, larvae infected with Mab fLuc were injected with 10 µL of 10% D-luciferin sodium salt (1.25 mg/mL in PBS, GoldBio, USA) into the haemocoel five minutes prior to image acquisition. *In vitro, in vivo* and *ex vivo* imaging was performed using the IVIS spectrum imaging system (Revvity, USA), capturing five consecutive images per scan (30 seconds exposure, 13.4 cm FOV, 0.5 cm subject height, open emission filter, no overlay). Regions of interest (ROI) were placed on each well and the peak total photon flux from the serial acquisitions (photons/second) was used for analysis. Data were analyzed using Living Image Software version 4.5.4 (Revvity, USA).

### 2.4 Colony-forming units

To determine the absolute viable cell count in the inocula and larval homogenates for BLI-CFU agreement evaluation, CFU quantification was carried out on the freshly prepared inoculum and on larval homogenates one hour post infection. Larvae were first imaged by *in vivo* BLI and were subsequently sacrificed immediately after imaging, enabling direct correlation analysis between luminescent signal and viable bacterial burden. For CFU determination, 10-fold serial dilutions were prepared, and 25 µL of each dilution were plated on 7H10 agar plates and incubated at 37 °C. Colonies were counted after seven days to allow all viable bacteria to form countable colonies to avoid underestimation of bacterial burden. Data is expressed as CFU/mL.

### 2.5 Antimycobacterial treatment

Rifabutin (RFB, TCI Chemicals, Japan) solution was diluted in PBS containing 10% DMSO to obtain final doses of 100, 50 and 5 mg/kg. Posaconazole (PCZ, Noxafil, MSD, USA) solution was diluted to obtain a final dose of 4 mg/kg in 0.9% sterile saline, and served as a negative control for antimycobacterial efficacy. Based on an average larval weight of 300 mg, treated and sham-treated larvae received a total injection volume of 10 µL of either rifabutin, posaconazole or vehicle (PBS containing 10% DMSO). Treatments were freshly prepared and administered by injection into the last proleg, starting from one hour post infection and repeated from day one to day four post infection.

### 2.6 Statistical analysis

All statistical analyses were performed using GraphPad Prism version 8.0.2. The Pearson correlation coefficient (r) was computed to evaluate data agreement. Log_10_-transformed longitudinal BLI data and health scoring were analyzed using a two-way repeated measures ANOVA with the Geiser-Greenhouse correction, to test for significant differences between inocula and longitudinal treatment effects. When missing values occurred due to larval death, analyses were performed using a mixed-effects model. Missing longitudinal BLI measurements due to larval death were handled using the last observation carried forward (LOCF) approach, and these values were included in graphical and statistical analyses. Survival data were analyzed using Kaplan-Meier estimates, and group differences were assessed using the Mantel-Cox long-rank test. The results were considered statistically significant when p < 0.05.

## 3 Results

### 3.1 Luminescence stability of Mab fLuc and Mab Lux ensure reliable larval imaging over time

To determine whether our bioluminescent Mab reporters enable reliable and stable *in vivo* larval imaging, we first assessed the temporal stability and sensitivity of photon emission (luminescence) during the scanning timeframe. Establishing stable signal kinetics is essential to define when imaging can be performed and to ensure that observed differences are not driven by signal decay. Luminescence was therefore monitored over time one hour after infecting *G. mellonella* larvae with inocula ranging from 10^2^ to 10^7^ CFU for Mab fLuc and 10^2^ to 10^8^ CFU for Mab Lux. Mab fLuc infected larvae were imaged sequentially for 20 minutes following D-luciferin administration to characterize substrate-dependent signal kinetics. Larvae infected with Mab Lux, which produces light autonomously and does not require exogenous substrate, were monitored for 10 minutes. This comparison allowed us to evaluate the temporal stability of luminescence for each strain and to assess their suitability for consistent *in vivo* larval imaging.

**Figure 1:**
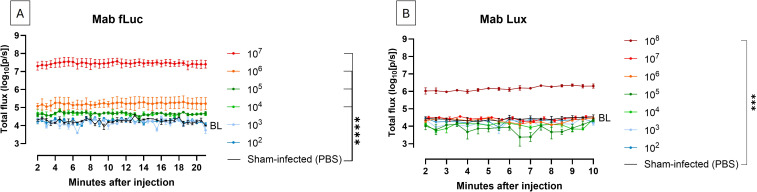
Luminescent signal remains stable over time following infection. **(A)** *In vivo* BLI of *G. mellonella* larvae infected with Mab fLuc at inocula ranging from 10² to 10^7^ CFU, monitored for 20 minutes at 1 hour post infection. BL (baseline) represents background signal (sham-infected larvae). **(B)** 10 minutes *in vivo* BLI scan of infected *G. mellonella* larvae with Mab Lux inocula ranging from 10^2^ to 10^8^ CFU at 1 hour post infection. Bars indicate mean ± SD for each group (n = 3). Statistical significance: ***P < 0.001, ****P < 0.0001.

Mab fLuc exhibited a stable bioluminescent signal as early as two minutes after D-luciferin injection, which remained stable for at least 20 minutes, well beyond the average imaging duration, which lies below ten minutes (**Figure 1A**). Importantly, BLI of Mab fLuc allowed clear visual discrimination between sham-infected larvae and larvae infected with an inoculum as low as 10^5^ CFU within one hour post infection (**Figure 1A**). Mab Lux produced a stable signal for at least 10 minutes but displayed markedly lower sensitivity at this early time point, with detectable luminescence only at the highest inoculum tested (10^8^ CFU) (**Figure 1B**). Together, these results confirm that both strains exhibit sufficient luminescence stability to support reliable larval imaging, with Mab fLuc enabling more sensitive detection of lower mycobacterial burdens one hour post infection compared to Mab Lux.

### 3.2 Emission spectrum characterization reveals improved *in vivo* detectability of red-shifted Mab fLuc

We characterized the emission spectra of Mab fLuc and Mab Lux in *Galleria mellonella* to determine their peak wavelengths and relative signal output. Defining the emission profiles of both reporter systems is important for confirming their suitability for *in vivo* imaging, as emission wavelength influences tissue penetration and signal detectability.

**Figure 2:**
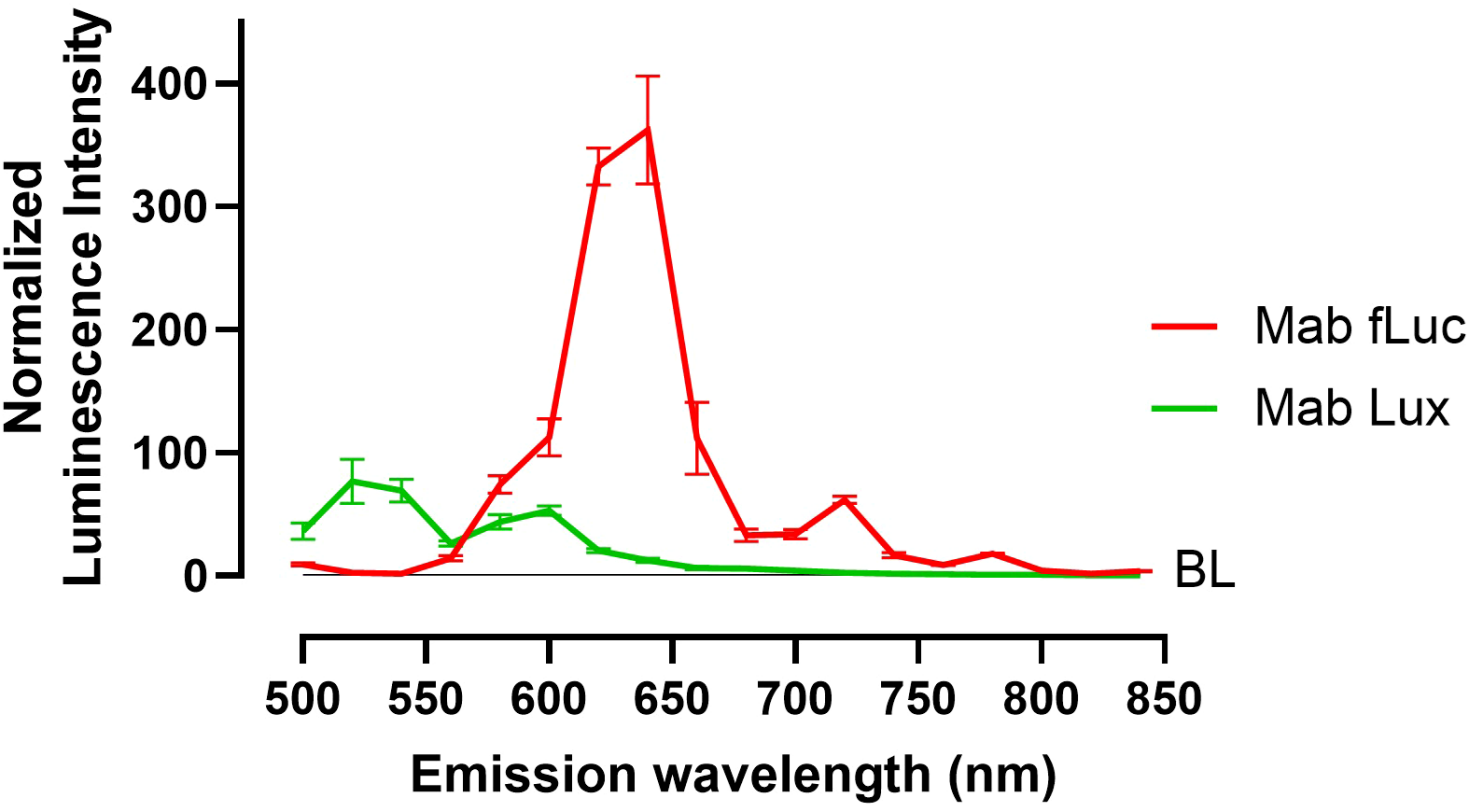
Emission spectra of Mab fLuc and Mab Lux correspond to the red and green spectrum, respectively. Spectral scan of *G. mellonella* larvae infected with 10^7^ CFU Mab fLuc and 10^8^ CFU Mab Lux at 1 hour post infection. BL = baseline (larvae sham-infected with PBS). Luminescence intensity normalized to baseline. N = 3 per group.

Mab fLuc, which incorporates a red-shifted firefly luciferase, exhibited a peak emission at 640 nm, within the red spectrum (**Figure 2**). Mab Lux, which lacks the red-shifted firefly luciferase, showed peak emission at 520 nm, corresponding to the green spectrum (**Figure 2**). Importantly, the red-shifted emission of Mab fLuc was associated with a higher detected signal intensity under *in vivo* imaging conditions, indicating an improved photon yield compared to Mab Lux, and supporting its preferential use for imaging deep-seated infections, including those in the lungs of mice.

### 3.3 Real-time bioluminescence imaging provides a quantitative readout of mycobacterial burden *in vitro, in vivo* and *ex vivo*

To determine whether BLI can serve as a complementary quantitative approach to conventional CFU analysis, we evaluated the correlation between BLI signals relative to mycobacterial burden across a defined dynamic range. To this end, predefined inocula with known CFU concentrations were used and subsequently validated by plating.

**Figure 3:**
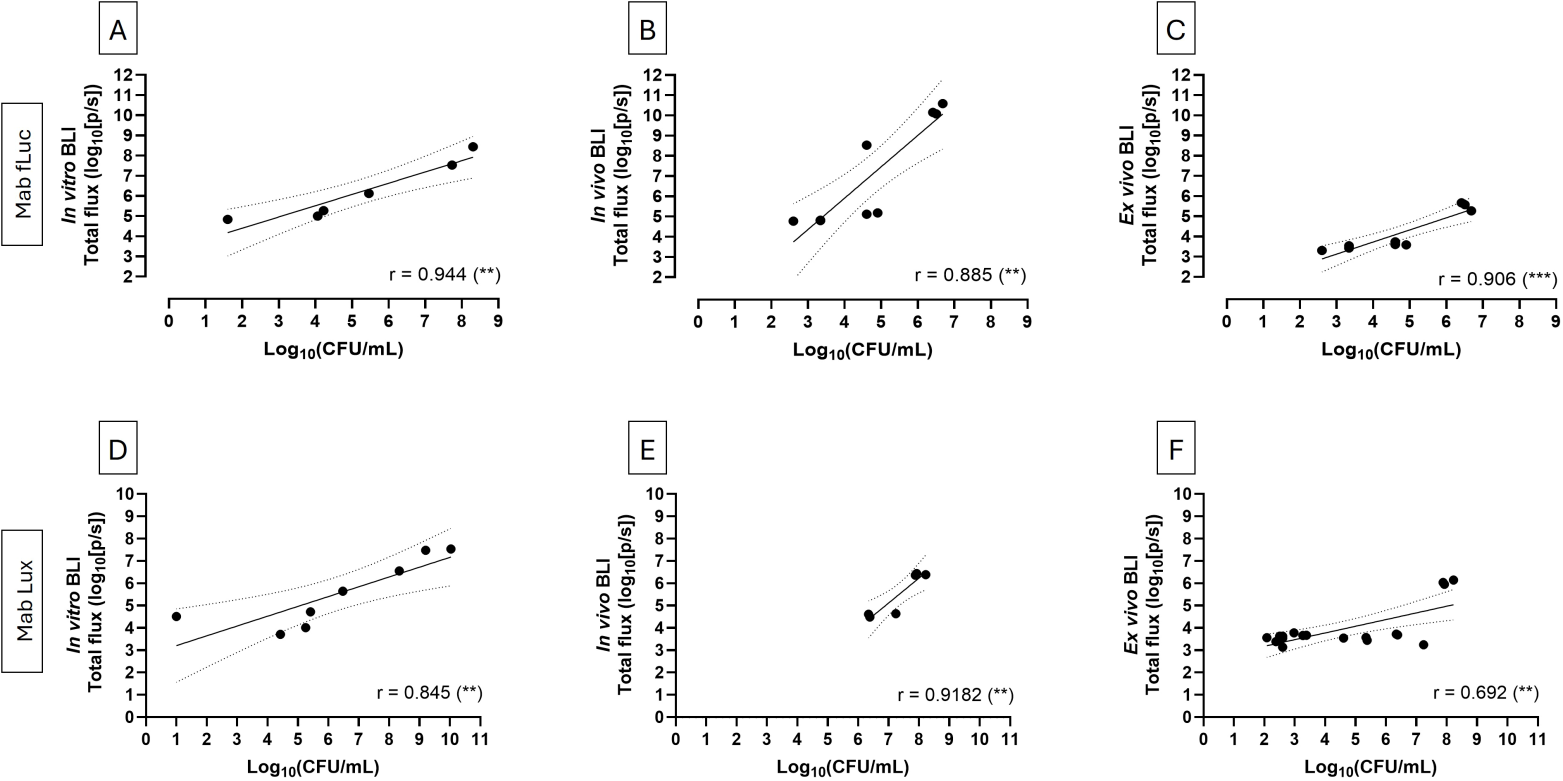
*In vitro, in vivo*, and *ex vivo* BLI signals show a reliable agreement with corresponding CFU counts. Correlation between BLI signals and CFU counts for Mab fLux **(A–C)** and Mab Lux **(D–F)**. **(A, D)** *In vitro* BLI of mycobacterial inocula ranging from 10^2^ to 10^7^ CFU. **B,E**) *In vivo* BLI of *G. mellonella* larvae at 1 hour post infection across the same inoculum range. **C, F**) *Ex vivo* BLI of larval homogenates at 1 hour post infection, with corresponding CFU counts. Both BLI signals and CFU values were log_10_-transformed prior to Pearson correlation analysis. r = Pearson correlation coefficient. Statistical significance: **P < 0.01, ***P < 0.001.

BLI signals obtained under each condition were correlated with the corresponding CFU counts. *In vitro* BLI of mycobacterial inocula ranging from 10^2^ to 10^7^ CFU correlated strongly with CFU counts (**Figures 3A, D**). Similarly, *in vivo* BLI signal intensity measured in infected *G. mellonella* larvae one hour post infection correlated well with CFU counts obtained from the larval homogenates immediately after imaging (**Figures 3B, E**), This correlation was further confirmed by *ex vivo* BLI analysis of corresponding larval homogenates for both strains (**Figures 3C, F**). Overall, log_10_-transformation of both BLI signal intensities and CFU counts revealed strong linear correlations across all experimental conditions in both strains. However, differences in sensitivity between the strains were evident at lower inoculum levels. At one hour post infection, the fLuc strain produced a detectable signal above baseline *in vivo* at 10^5^ CFU, whereas for Mab Lux, signal detection above baseline was only observed at 10^7^ CFU (**Figures 3B, E**). Together, these consistent linear relationships demonstrate that BLI provides a reliable quantitative surrogate of mycobacterial burden. Importantly, unlike CFU plating, *in vivo* BLI enables real-time, longitudinal monitoring of infection dynamics, eliminating the prolonged delays and the need for terminal endpoint sampling associated with CFU plating, and providing a faster and less labor-intensive approach for assessing infection progression.

### 3.4 BLI outperforms health scoring and survival in discriminating mycobacterial inocula

Sensitive discrimination between mycobacterial inocula is essential for evaluating treatment efficacy. To assess the power of BLI to discriminate different NTM inocula, we assessed the sensitivity of BLI and compared it to traditional readouts like health scoring and survival. Larvae were infected with 10^2^, 10^5^ to 10^8^ CFU of either Mab fLuc or Mab Lux, and monitored for four days post infection using BLI, health scoring and survival.

**Figure 4:**
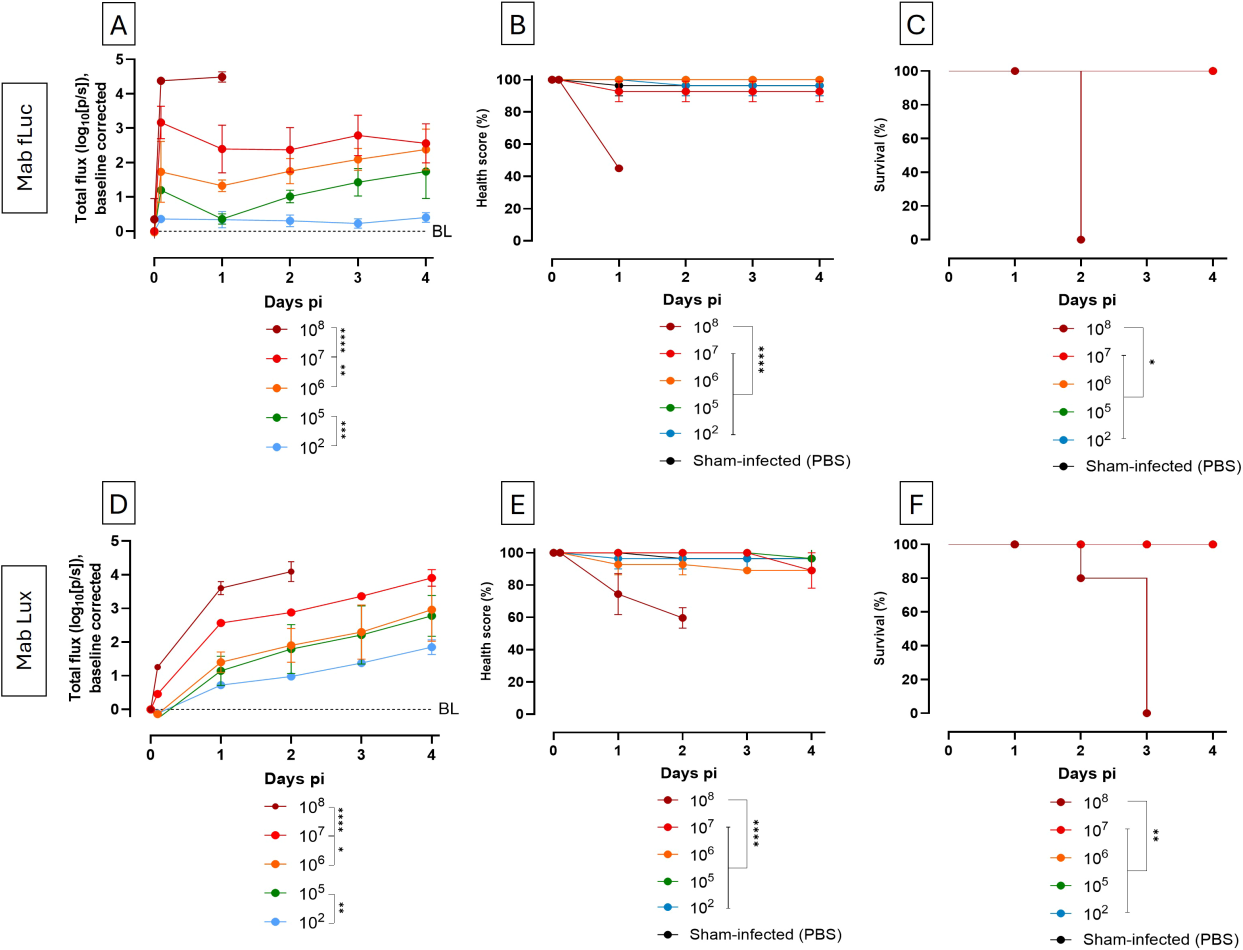
*In vivo* BLI accurately detects early differences in mycobacterial inocula levels not captured by health scoring or survival. **(A, D)** Longitudinal *in vivo* BLI of *G. mellonella* larvae infected with Mab fLuc **(A)** or Mab Lux **(D)** at inocula ranging from 10^2^ to 10^8^ CFU over 4 days post infection. Signals are expressed as log_10_ total flux (photons/s- and normalized to BL, baseline, defined as the signal from sham-infected larvae injected with PBS. **(B, E)** Corresponding health scores and **(C, F)** survival over the same time period. Data represent mean ± SD (n = 3 larvae per group). Statistical significance was determined relative to sham-infected controls. *P < 0.05, **P < 0.01, ***P < 0.001, and ****P < 0.0001.

*In vivo* BLI enabled clear and quantitative discrimination between inocula as early as one hour post infection for Mab fLuc, with bioluminescence signal intensity increasing proportionally to bacterial burden (**Figure 4A**). By one day post infection, all tested inocula were distinctly separated, with a limit of detection of 10^2^ CFU, confirming the high sensitivity of Mab fLuc. For Mab Lux, luminescence increased gradually over time, indicating a more pronounced Mab proliferation compared to Mab fLuc, with evident discrimination between inocula from one day post infection onward (**Figure 4D**). Even the lowest inoculum (10^2^ CFU) produced a detectable signal above baseline from one day post infection, with signal intensity increasing over time. However, early differentiation between inocula at one hour post infection was not possible, which is consistent with observations in **Figure 1A**.

In contrast, health scoring and survival failed to detect differences between inocula over all time points during the observation period and only indicated severe infection at the highest dose (**Figures 4B, C, E, F**). Although BLI indicated a progressive increase in bacterial burden over time, larval health remained on average above 90%, failing to capture an increased bacterial burden until the burden was too high. Differences in health score or survival may become apparent at later timepoints, albeit that longer health observation periods may be limited by the onset of pupation.

Together, these observations indicate that BLI significantly outperforms traditional readouts, enabling early, quantitative, and dose-dependent direct longitudinal measurement of mycobacterial burden and proliferation in real-time, within an experimentally accessible timeframe.

### 3.5 Early antibiotic treatment effects are detectable by BLI but not by conventional readouts

**Figure 5:**
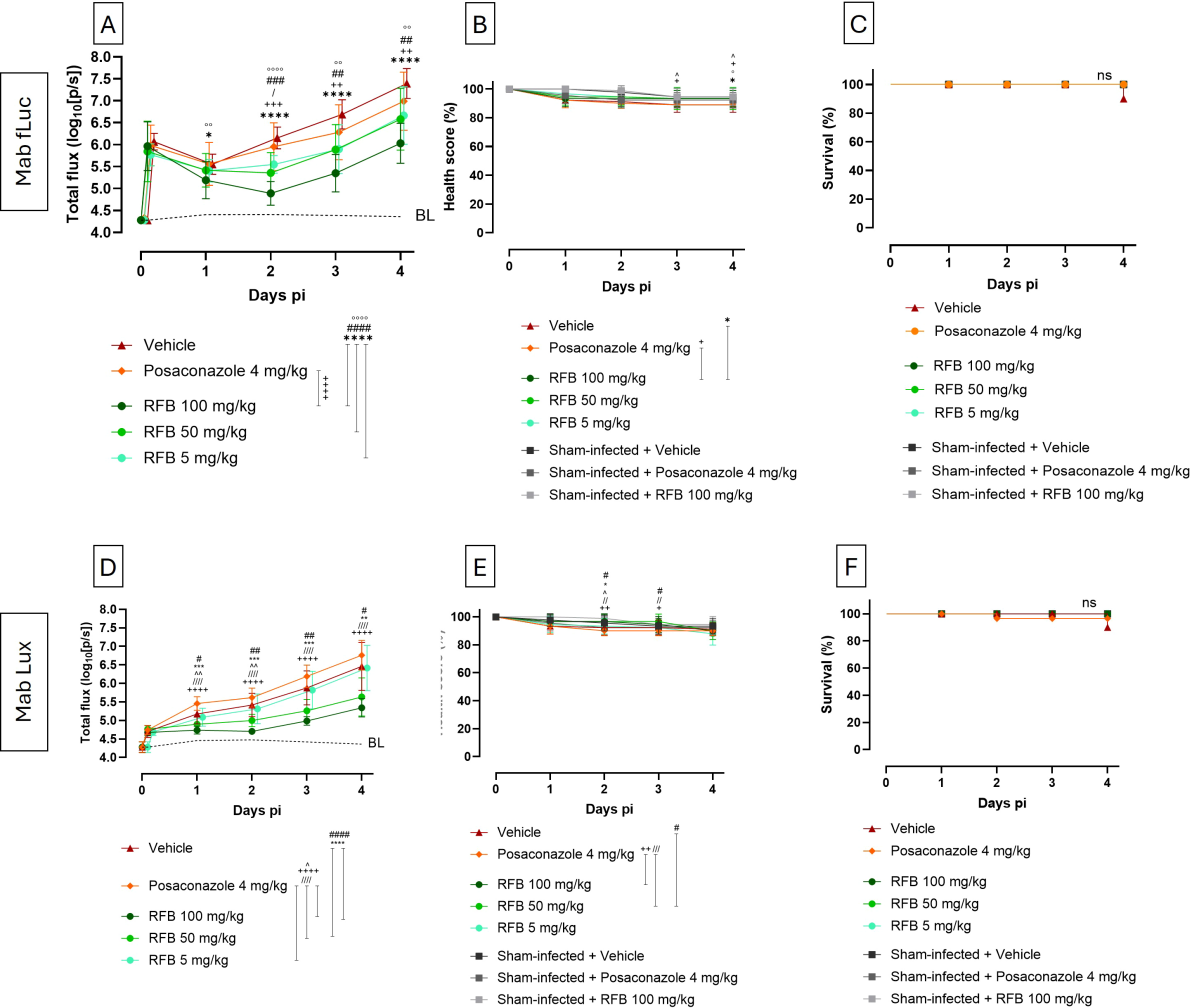
*In vivo* BLI captures early treatment efficacy, whilst health score and survival do not. **(A)** *In vivo* BLI signal of larvae infected with 10^6^ CFU Mab fLuc and **(D)** *in vivo* BLI signal of larvae infected with 10^7^ CFU Mab Lux over 4 days post infection. BL (baseline) represents background signal (sham-infected larvae treated with PBS). RFB, rifabutin, vehicle = PBS + 10% DMSO. **(B, E)** health score and **(C, F)** survival. Statistics in the legends refer to pairwise longitudinal differences over 4 days, and statistics on the graph refer to differences at individual days: “*” between RFB 100 mg/kg and vehicle, “#” between RFB 50 mg/kg and vehicle, “°” between RFB 5 mg/kg and vehicle, “+” between RFB 100 mg/kg and posaconazole, “/” between RFB 50 mg/kg and posaconazole, “^” between RFB 5 mg/kg and posaconazole. Bars indicate mean ± SD for each group (n = 10 in all groups). Statistical significance: *P < 0.05, **P < 0.01, ***P < 0.001, ****P < 0.0001.

Next, we set out to assess the ability of BLI to sensitively detect treatment effects, compared to health scoring and survival analysis. We infected larvae with optimized inocula of Mab fLuc (10^6^ CFU) and Mab Lux (10^7^ CFU), as these doses produced a steady increase in BLI signal over time without significantly affecting larval health, as shown in **Figures 4A, D**. Larvae were treated daily with different doses of rifabutin (RFB) for four days. Posaconazole and PBS served as negative and vehicle controls, respectively. All treatments were first assessed for toxicity by injecting these treatments daily for four days in sham-infected larvae, and any toxicity could be excluded (**Figures 5B, C, E, F**). BLI was able to detect significant, dose-dependent decreases in bacterial burden as early as one day post infection compared to control groups, even though luminescence kept increasing gradually over time in both groups across the different inocula (**Figures 5A, D**). In contrast, health scoring and survival only showed minimal to non-treatment effects. For Mab fLuc, a significant difference in health score was observed only at the highest dose of rifabutin (100 mg/kg) when compared to posaconazole and vehicle-treated groups (**Figure 5B**), while no differences in survival were detected (**Figure 5C**). Similarly, for Mab Lux, significant differences in health scores were only observed between rifabutin at 100 and 50 mg/kg versus posaconazole, as well as between rifabutin at 50 mg/kg and vehicle treatment (**Figure 5E**), with survival remaining unaffected across all treatment groups (**Figure 5F**). Notably, no difference was detected between the lowest rifabutin dose (5 mg/kg) and the sham-treated controls using these conventional readouts, despite clear effects being observed with BLI. In conclusion, these findings demonstrate that *in vivo* BLI outperforms health scoring and survival in detecting both early and dose-dependent antibiotic effects, highlighting its value over traditional endpoints for therapeutic evaluation in *G. mellonella*.

## 4 Discussion

In this study, we present a standardized and quantitatively validated bioluminescent *Galleria mellonella* infection model enabling sensitive, real-time, and longitudinal quantification of *Mycobacterium abscessus* infection. BLI proved to be a rapid, non-invasive and quantitative readout that strongly correlated with CFU counts, reduced variability and outperformed traditional *G. mellonella* longitudinal readouts like health scoring and survival. This model allowed for a dynamic early differentiation of inoculum doses and detected dose-dependent treatment effects as early as one day post infection with rifabutin, enabling monitoring over multiple days. These findings not only establish BLI as a sensitive tool within the *G. mellonella* model but also highlight *G. mellonella* itself as a valuable complementary preclinical screening tool for novel antimycobacterial compounds.

We employed two bioluminescent Mab strains to compare two different strategies (reporter systems) for bioluminescence imaging. The Mab fLuc strain expresses a red-shifted firefly luciferase requiring substrate administration, resulting in deeper tissue imaging and higher photon flux. In mammalian systems, red-shifted emission is less absorbed by hemoglobin, improving tissue penetration and reducing light scattering significantly (Liang et al., 2012; Vanherp et al., 2019). Indeed, Mab fLuc produced a higher *in vivo* signal intensity than Mab Lux, underscoring its suitability for murine imaging applications. In contrast, Mab Lux emits light in the green spectrum, which is more scattered and readily absorbed by hemoglobin, but its luxCDABE operon enables autonomous light production without substrate administration (Bento et al., 2024). This simplifies longitudinal imaging and is especially beneficial for multi-dose therapies by reducing handling and injection-related stress in larvae.

Interestingly, although a previous study reported that only Mab fLuc produced quantifiable luminescence *in vivo*, we successfully detected quantifiable luminescence from Mab Lux in *G. mellonella* (Bento et al., 2024). Additionally, with both strains we were able to discriminate mycobacterial inocula as early as one day post infection, and as low as 100 CFU. These findings highlight the flexibility of our model, allowing researchers to select the most appropriate strain depending on the experimental context: Mab Lux is well-suited for *G. mellonella* studies because of the minimal required injections, while Mab fLuc is more appropriate for murine studies as tissue penetration can be enhanced: an approach planned for future studies.

Our bioluminescent *G. mellonella* model overcomes key limitations of CFU-based quantification. CFU analysis is labor-intensive, variable, restricted to endpoint measurements, and delayed by the slow growth of Mab colonies, which require up to seven days of incubation (Vanherp et al., 2019; Vanhoffelen et al., 2023). Here, BLI provides real-time, dynamic monitoring of infection progression. Pearson correlation analyses confirmed that BLI reliably reflects mycobacterial burden across a broad dynamic range for both strains. Moreover, BLI outperformed health scoring and survival, which provide binary outputs and are prone to interobserver variability. Our data shows that BLI can distinguish between infection levels and treatment groups as early as one day post infection, much earlier and with greater sensitivity compared to these traditional readouts (Vanhoffelen et al., 2023). A limitation of BLI, however, is that complete bacterial eradication cannot be detected below the luminescence threshold, necessitating CFU-based confirmation at endpoint. Thus, BLI and CFU analyses are complementary when testing efficacy of novel compounds.

This model can potentially help to improve the translation from *in vitro* to *in vivo* efficacy studies, a known bottleneck in antimicrobial development. Many compounds that show promise *in vitro* fail in mammalian models. Intermediate models like *G. mellonella* may serve as a valuable bridge, enabling early *in vivo* efficacy screening before advancing to more complex and costly and ethically constrained mammalian studies (Meir et al., 2018; Hosoda et al., 2020; Theuretzbacher et al., 2020; Curtis et al., 2022). Moreover, as currently no good Mab mouse model is available, we are offering a much-needed intermediate *in vivo* tool to support early efficacy screening.

The ability to assess both toxicity, maximum tolerated doses, and early efficacy within a single experiment further enhances the utility of this model for early-phase drug development. *G. mellonella* has been successfully used to evaluate monotherapies and drug combinations, including antifungal studies demonstrating synergy between posaconazole and tacrolimus against *Aspergillus fumigatus* (Vanhoffelen et al., 2025). Moreover, virulence and toxicity of bacterial strains in *G. mellonella* translate well to murine models, as is demonstrated in previous studies involving multiple bacterial species (Vanhoffelen et al., 2024). Additionally, antibiotic efficacy and dosing in *G. mellonella* have also been shown to correlate with human clinical data, further supporting its translational relevance (Ignasiak and Maxwell, 2017; Vanhoffelen et al., 2024).

Here, we demonstrated the feasibility of using *G. mellonella* for preliminary toxicity screening, as shown in our evaluation of posaconazole and rifabutin, and to assess initial antimycobacterial efficacies. These findings highlight the value of our bioluminescent *G. mellonella* model as a complementary powerful and sensitive tool to support future identification of novel, potent, and synergetic therapeutic drugs for controlling Mab infection.

Despite its advantages, the *Galleria mellonella* model has limitations that need to be considered. Larvae lack an adaptive immune system and a comparable respiratory system to mammals, which restricts its ability to fully mimic host-pathogen and broncho-alveolar surface interactions seen in vertebrates (Sheehan et al., 2018). Furthermore, pharmacokinetic and pharmacodynamic properties in larvae differ from those in mammals, which may affect drug absorption and metabolism. These differences highlight the importance of using *G. mellonella* as an intermediate model in the preclinical drug development pipeline, but also indicate the continued importance of validation in mammalian models (Vanhoffelen et al., 2023; Vanhoffelen et al., 2024).

In conclusion, we successfully established a bioluminescent *Galleria mellonella* infection model as a robust, real-time, quantitative, and longitudinal platform to assess *Mycobacterium abscessus* burden and treatment responses. By integrating BLI in this model, we introduce a sensitive and scalable *in vivo* readout that strengthens the role of *G. mellonella* as a translational prescreening step within the preclinical drug development pipeline. This approach helps bridging the gap between *in vitro* and *in vivo* testing by enabling early, quantitative evaluation of drug efficacy in a living host, thereby improving the predictive power of early-phase drug selections. This model contributes to reducing reliance on mammalian models, while still enabling robust and quantitative measurements of infection and treatment efficacies of novel compounds (Vanhoffelen et al., 2024). As visualized in the graphical abstract (André, 2026), this model enables a fully BLI-driven screening pipeline, combining rapid *in vitro* assessment using plate-based luminescence readouts, with quantitative *in vivo* evaluation in *G. mellonella*, followed by validation in murine infection models. Incorporation of this model allows preclinical pipelines to be accelerated, facilitating a more informed selection of candidate compounds before progression to murine studies.

## Data Availability

The raw data supporting the conclusions of this article will be made available by the authors, without undue reservation.
